# Intervertebral Disc Tissue Engineering Using Additive Manufacturing

**DOI:** 10.3390/gels9010025

**Published:** 2022-12-29

**Authors:** Minami Yoshida, Paul Richard Turner, Jaydee Dones Cabral

**Affiliations:** 1 Centre of Bioengineering & Nanomedicine, Department of Oral Rehabilitation, University of Otago, Dunedin 9054, New Zealand; 2 Department of Microbiology & Immunology, University of Otago, Dunedin 9054, New Zealand

**Keywords:** intervertebral disc, additive manufacturing, tissue engineering, 3D printing, biomaterials, hydrogels, stem cells

## Abstract

Intervertebral disc (IVD) degeneration is one of the major causes of lower back pain, a common health condition that greatly affects the quality of life. With an increasing elderly population and changes in lifestyle, there exists a high demand for novel treatment strategies for damaged IVDs. Researchers have investigated IVD tissue engineering (TE) as a way to restore biological and mechanical functions by regenerating or replacing damaged discs using scaffolds with suitable cells. These scaffolds can be constructed using material extrusion additive manufacturing (AM), a technique used to build three-dimensional (3D), custom discs utilising computer-aided design (CAD). Structural geometry can be controlled via the manipulation of printing parameters, material selection, temperature, and various other processing parameters. To date, there are no clinically relevant TE-IVDs available. In this review, advances in AM-based approaches for IVD TE are briefly discussed in order to achieve a better understanding of the requirements needed to obtain more effective, and ultimately clinically relevant, IVD TE constructs.

## 1. Introduction

Back pain statistics are stark; more than 80% of the adult population is affected by lower back pain, causing a huge socioeconomic burden, especially in developed countries [1,2]. Forty percent of this lower back pain is associated with intervertebral disc (IVD) degeneration, which manifests in 97% of the population greater than 50 years old [3].

**Figure 1:**
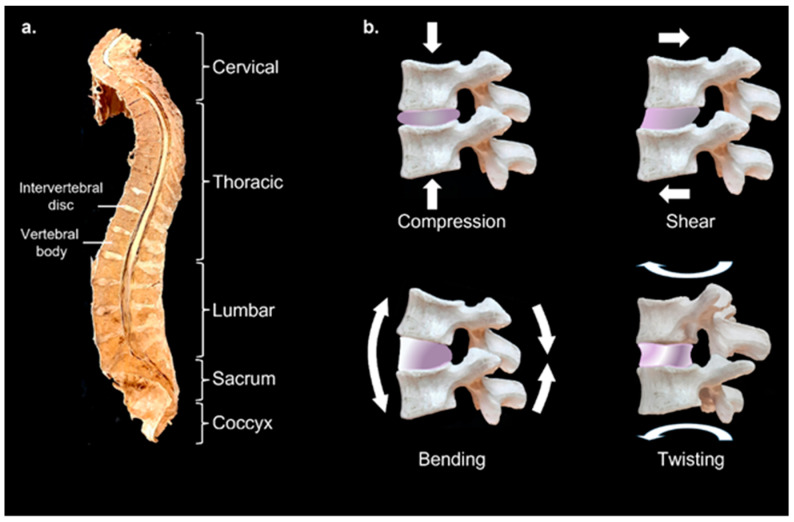
The human spine and intervertebral discs. (**a**) Median sagittal section of human spine showing white intervertebral discs between vertebral bodies. (**b**) Types of movement and forces intervertebral discs need to endure. Images prepared using material from the University of Otago, Anatomy Museum.

Healthy IVDs serve several purposes and are uniquely structured to perform them. They cushion the stacked vertebrae and so must be resistant to compressive load, but still be flexible enough to allow spinal movement. They must possess good viscoelastic properties and function reliably over a lifetime, Figure 1.

**Figure 2:**
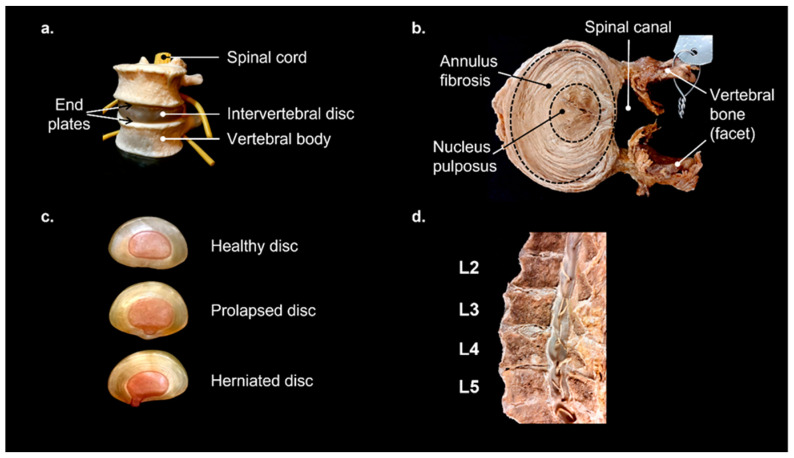
Intervertebral discs. (**a**) Model showing intervertebral disc between two adjacent vertebrae, nerves and spinal cord shown in yellow. (**b**) Transverse section through a human intervertebral disc showing the lamellae of the annulus fibrosis and the central nucleus pulposus. (**c**) Transverse section models of progression of intervertebral disc degeneration, from healthy to prolapsed to herniated. (**d**) Sagittal section of lumbar region of human spine, with vertebrae labelled. The nucleus pulposus of the L3/L4 disc is clearly visible as a white oval structure. However, the disc between L4 and L5 is severely degenerated resulting in vertebral bodies in direct contact with one another. Sections were from a 78-year-old female prepared using material from the University of Otago, Anatomy Museum.

To meet these demands, the IVD consists of three parts; an internal type II collagen and proteoglycan-rich compartment (nucleus pulposus, NP), a fibrous outer part (annulus fibrosus, AF) composed of repeating layers of type I and II collagen-rich sheets (lamellae), and the cartilaginous end plates (CEPs) that are bound to both the vertebrae and disc and control nutrient import [4], Figure 2.

Intervertebral disc degeneration (IVDD) is a broadly identified cause of back pain which exhibits increased proinflammatory activity and extracellular matrix (ECM) degradation leading to structural and biomechanical changes [5,6,7]. Risk factors for IVDD are age [8], genetics [9], smoking, and lifestyle factors such as diabetes, obesity and a history of injury.

Current treatment regimens for IVD degeneration and associated lower back pain involve mostly conservative treatments, such as altering one’s lifestyle, rehabilitation and taking medication. More aggressive surgical treatments include spinal fusion, discectomy and total disc replacement [10,11]. Surgical treatments are irreversible, therefore only applicable for patients with severely degenerated IVDs. Although the surgical methods do alleviate pain in 60 to 80% of patients, these are still not ideal as they can cause further issues such as acceleration of adjacent IVD degeneration, hyper-mobility, hypo-mobility, implant displacement and inflammatory reactions [12,13]. For these reasons, researchers and clinicians are exploring regenerative tissue engineering strategies to improve IVD treatment. Tissue engineering can provide not only the structural or mechanical restoration of the IVD, but also encourage the biochemical and cellular restoration of IVD tissue [14,15].

## 2. Regenerative Medicine

The regenerative medicine approach, using tissue-engineering scaffolds for IVD replacement, if successful, would be superior to current treatment strategies by alleviating pain, allowing short-term repair and long-term tissue regeneration, decreasing immune rejection and re-herniation, as well as preserving disc height and restoring the natural range of motion [15,16]. Over the last 20 years, researchers have successfully fabricated IVD constructs, with some focusing on the use of soft, injectable biomaterials to serve as carriers of cells and/or biomolecules to be delivered to the injury site; [7] however, none have managed to fully mimic the unique micro-scale architecture of native AF tissue. Although it is believed that utilizing biomaterials similar in composition to the natural ECM of native tissue would be ideal, combining two distinct fibrous and gelatinous tissues is problematic. Therefore, an adequate combination of biomaterials, fabrication methods, cells, and biochemical and mechanical factors must all be investigated for the development of successful IVD constructs [3].

The field of regenerative medicine is vast—a Medline search results in over 95,000 hits—and excellent reviews on the topic abound [17,18] with the incorporation of vasculature a key highlight [19].

Here, we review additive manufacturing technologies used for IVD tissue engineering, with a focus on the replacement of the damaged IVD as a whole.

## 3. Additive Manufacturing in IVD Tissue Engineering

Additive manufacturing, also known as rapid prototyping or solid free-form technique, forms 3D objects in a layer-by-layer manner to produce a three-dimensional structure based on computer-aided design (CAD) data [20,21]. Some of the major additive manufacturing technologies used for tissue engineering are fused deposition modelling (FDM), 3D bioprinting, selective laser sintering, stereolithography and melt electrowriting (MEW) [21,22]. The advantages of rapid prototyping are high efficiency, scaffold reproducibility and printability; and the ability to construct complex, biomimetic architectures and structures [23]. Additive manufacturing also allows researchers to control the mechanical and biological properties, as well as the biodegradation rate of the scaffold by controlling porosity and infill structure in addition to biomaterial selection [24].

More and more research facilities have started utilising additive manufacturing technologies for tissue engineering purposes. IVD tissue engineering is not an exception. The technique is highly advantageous to produce personalised IVD scaffolds, as IVD shapes are unique between individuals and IVDs have a highly complex architecture. In addition, rapid prototyping can integrate with imaging techniques, such as computed tomography scanning, to create scaffolds with a customised structure.

Although currently, a majority of the IVD tissue engineering studies utilise polymer electrospinning or hydrogel moulding, some of them have employed additive manufacturing technologies (Table 1). Composite structures using multiple AM techniques, such as 3D melt extrusion and 3D bioprinting together, along with growth factor (GF) delivering nanoparticles (NPs) encapsulated within the bioink are used to recreate IVD complexity [25].

Three-dimensional printing is an extrusion-based, additive manufacturing technique that extrudes ink/bioink through a nozzle in a layer-by-layer manner to create 3D structure. It includes FDM and 3D bioprinting, which employ different ways of preparing ink (e.g., polymer solutions, molten thermoplastic polymers and cell-laden hydrogels) for extrusion (Table 2).

FDM is used in tissue engineering due to its affordability and ease of scaffold manufacturing [33]. Unlike other additive manufacturing methods, FDM does not require any solvents and uses easy-to-handle materials [34]. The FDM process starts by slicing the 3D CAD data into layers. Then, the filaments or pellets of the thermoplastic materials are heated to extrude through the nozzle in specific configurations specified by the CAD model. The molten polymers are deposited in a layer-by-layer manner, fusing each layer to the previously printed layers [33]. The extrusion nozzle continues to move in the horizontal x-y plane while the build platform moves vertically down [35]. To print scaffolds with highly complex structures, the use of water-soluble support material is common. After printing is complete, the support materials are removed [35]. FDM is more common and inexpensive compared to selective laser sintering. Scaffolds fabricated with FDM technology tend to have high precision and mechanical strength [20]. The raw materials it can handle are limited to thermoplastic polymers, however, such as polycaprolactone (PCL), polylactic acid (PLA), acrylonitrile butadiene styrene, polyester, and polycarbonate. Encapsulation of other polymers in these materials has also been investigated for tissue engineering purposes [36].

For tissue engineering the whole IVD, FDM is also commonly used, mainly to provide mechanical strength to withstand the physical loads that are expected to be applied to the IVD scaffolds. The biomaterials used in this field are PCL, PLA, and FlexiFil PLA (FPLA) filaments [24,25,26,27,28,29,30]. The scaffolds created with the FDM technique have been used as CEP, AF region, structural support for the IVD, or for the whole IVD with different infill density between AF and NP regions. Other studies also employed FDM indirectly to create moulds to crosslink hydrogels to make IVD scaffold solely out of hydrogels, or to produce angular patterns simulating the collagen fibre orientation of the AF [37,38].

3D bioprinting is a type of 3D printing which uses hydrogels that encapsulate cells as a bioink. The printed scaffold is typically cured/crosslinked using UV light or other chemicals after the printing is complete. Similar to FDM and other extrusion-based 3D printing, the bioink is extruded through a nozzle and deposited onto a platform that typically, is cooled [23]. Some of the advantages of 3D bioprinting are that it allows zone-specific distribution of cells, and the encapsulated cells have high cell viability [20]. Although hydrogels tend to have insufficient mechanical properties for use in IVD tissue engineering, some studies have presented sufficient results utilising double network crosslinking or the addition of nanofibers [2,29].

Several studies bioprinted cell-laden hydrogels such as alginate, gellan gum–poly (ethylene glycol) diacrylate (GG–PEGDA), and gelatin–hyaluronic acid–sodium alginate seeded with chondrocytes and bone marrow stromal cells [24,25,26,29,30]. In these studies, hydrogels were used as part of the NP along with FDM scaffolds. The use of hydrogels generally increases the biocompatibility of the scaffolds and enhances cell adhesion and proliferation, hence it is a valid method for fabricating large tissue constructs such as IVDs [25].

Melt electrowriting is a newly emerging technique, that combines the best of both worlds of 3D printing and electrospinning. It deposits electrically controlled fine microfibres onto the platform to fabricate precise layer-by-layer scaffolds. The main feature of MEW is that it uses heat-molten biomaterials, but with much finer strands deposited compared to FDM. MEW also allows control over the microarchitecture of the scaffold unlike electrospinning [39]. This is particularly good for mimicking the microarchitecture of the native AF tissue. Melt electrowriting was also used in one of our previous studies [39] with the aim of using an oriented microfibrous structure as an AF scaffold. Future research will examine the characteristics of the rudimentary IVD produced.

**Table 2:** Advantages and disadvantages of additive manufacturing technologies used for whole IVD tissue engineering.

AM Technology	Advantages	Disadvantages
3D bioprinting	High cell viability; able to encapsulate cells, growth factors and nutrients; zone-specific distribution of cells.	Limited structural integrity; limited mechanical strength.
Fused deposition modelling	Able to fabricate scaffolds with various porosity; cost effective; minimum waste [40].	Limited biomaterial range as ink; high temperature; potential exposure to toxic fumes.
Melt electrowriting	High control over scaffold microarchitecture with microfibers.	High temperature; limited biomaterial range as ink.
SLS	Reliable, fast, requiring no support structures with excellent mechanical properties.	Limited material selection, high shrink rate, higher waste than other AM techniques.

## 4. Future Perspectives and Challenges of Utilising Additive Manufacturing in Whole IVD Tissue Engineering

Despite the gross anatomy of the IVD appearing simple, reproduction of the complex microstructure has proven to be challenging [41]. While numerous studies have been performed and significant progress has been made in the last 20 years, there are still challenges and limitations that need to be addressed for clinically relevant IVD constructs [3]. Currently, the best biomimetic materials and fabrication methods for fabricating IVD constructs are still under investigation. The ideal fabrication method is likely to result in a method that allows the fabrication of scaffold that has gradual transition of the structure and biochemical components from the outer AF to the inner AF, and then the NP region. Other microfabrication methods have been used in attempts to make complex, porous, tissue-engineered scaffolds, such as 3D projection stereolithography [42], and inkjet printing [43], to best replicate complex anatomic shaping and well-defined pore architecture [44]; however, an IVD construct with this level of microscopic or nanoscopic structural detail has not been constructed to date.

One of the main issues with IVD tissue engineering is that the rate of IVD regeneration is extremely slow [45]. This implies that in order to properly regenerate IVD through total disc replacement with tissue-engineered IVD, the scaffold needs to maintain its mechanical strength while slowly replacing itself with newly generated tissue. To allow this, the scaffold must degenerate at the same rate as the new tissue is formed. PCL, FPLA and polyurethane, the polymers used for whole IVD tissue engineering as mentioned above, have extensive degeneration rates of 1–2 years, more than 34 weeks, and 5–6 months, respectively [1,28,46]. It is worth noting that the scaffolds made with PLA became brittle after 26 weeks [28].

Developing scaffolds which mimic the microstructure of the native IVD is a good way to ensure that the ECM deposited is similarly aligned to that of native IVDs. This also encourages cells in the AF region to elongate in the appropriate direction, mimicking the native IVD environment. Jungst et al. reported that the differentiation of MSCs can be directed with MEW scaffold design, which suggests that additive manufacturing, in particular MEW, may provide great advantages in creating AF scaffold which mimics the native AF microstructure [47]. Another challenge with IVD tissue engineering is the optimum stabilisation method of tissue-engineered IVD. Scaffolds must be securely fixed in location so they will not slide out of the intervertebral disc space of individuals. Since all individuals have slight variations in the shape of IVDs, it is necessary that the fabricated IVD scaffolds have structure-specific constructs made fit to the individual. It can be hypothesised that fabricating CEPs with additive manufacturing technology using a 3D model derived from microcomputed tomography of the patient’s vertebrae might prevent tissue-engineered IVDs from dislocating. It may also be able to prevent proteoglycan loss during implantation, which is described later.

It is known that spinal fusion or total IVD arthroplasty can cause adjacent segment disease, a condition where the IVDs above or below the fused vertebrae degenerate post-surgery [48]. One of the main reasons for this is that the healthy IVDs above and below the operated segments are compensating for the lack of movement post-surgery, which causes unnatural strain on these IVDs [49]. In order to prevent the degeneration of adjacent native IVDs, it is crucial that the structural and mechanical properties of the tissue-engineered IVD matches that of the IVD it is replacing. The mechanical properties of the scaffolds fabricated using additive manufacturing methods seem to present high uniaxial compressive mechanical strength comparable to that of native IVDs [1,25,27]. Mallick et al. demonstrated that bone tissue scaffolds fabricated via 3D printing from hydroxyapatite (HA) and poly(vinyl)alcohol (PVOH) produced mechanically stable, porous scaffolds with compressive strength dependent on the rate of consolidation of surface pores and overall void volume [50]. Although further mechanical testing still needs to be carried out with more complex loadings closely mimicking the native environment, the ability of additive manufacturing to modify and control the microarchitecture of the scaffolds by altering polymer infill density, strand distance and porosity is a huge advantage to IVD tissue engineering [40].

Although there are other studies that performed “close-to-human” in vivo experiments for IVD constructs with large animals [38,51], to the authors’ knowledge there are no large-animal in vivo studies performed for IVD scaffolds fabricated using additive manufacturing technologies. As shown in Table 1, some studies have successfully demonstrated the scaffold viability in rat caudal disc spaces [29,30]; however, long-term in vivo experiments with large animals that have similar mechanical loads to humans is still a necessary step for IVD scaffolds made with additive manufacturing technologies. Additionally, the caudal discs of rats are much smaller in size compared to that of humans, suggesting the results obtained from these investigations might not be directly transferrable when scaled up to human size.

The IVD structure is the largest avascular structure in the human body. Therefore, it is prone to suffer from a lack of nutrients and cell diffusion throughout its scaffold [52]. However, an IVD scaffold developed with a PCL-based, nanofibrous AF with a small strand distance, still successfully demonstrated appropriate collagen, aggrecan, and cell diffusion throughout the scaffold in in vivo culture for 20 weeks [51,53]. This is encouraging because the fibre size of additive manufacturing techniques is much larger than that of electrospinning and thus allows for a higher rate of diffusion to enable cell survival.

Some studies reported the loss of proteoglycans from IVD scaffolds to culture medium in the in vitro environment during the 60 days of culture [54]. This loss of proteoglycan was also present in an in vivo experiment performed with beagle cervical discs [38]. Gullbrand et al. suggested that this proteoglycan loss could be prevented by including PCL foam CEPs prepared with a salt leaching technique in the IVD construct [51]. Sun et al. and Gloria et al. fabricated their IVD scaffolds including CEP made of PCL [25,27]. Sun et al. demonstrated that MSC differentiated to appropriate cell types and the glycosaminoglycans and collagen were deposited during 12 weeks of rat subcutaneous implantation, and no proteoglycan loss was reported [25]. This investigation further supports the hypothesis that the inclusion of CEP might prevent the loss of proteoglycans during in vivo/in vitro culture.

## 5. Conclusions

In recent years, there have been several studies exploring whole IVD tissue engineering using additive manufacturing. However, there are still several challenges to developing functional IVD constructs that have an optimal balance between the required structural, mechanical and biological properties. Due to the capability of fabricating patient-specific implants and easily customisable scaffolds, additive manufacturing technology is expected to gather more research interest in the field of tissue engineering. The addition of biomimetic CEP might resolve some of the issues associated with IVD tissue engineering. Further development and investigation of fabrication methods, biomaterials and cells are required for the future 3D success of IVD tissue engineering.

## Figures and Tables

**Table 1:** Fabrication methods, materials and key findings of whole IVD tissue engineering studies that used additive manufacturing technologies.

Fabrication Method	Material	Findings	Ref.
3D printing and lyophilisation	Degradable Polyurethane	Cells aligned along the concentric lamellae. Scaffold did not degrade after 19 days.	[1]
3D melt extrusion	PCL	Reconstructed IVD showed the same zone specific matrix as natural tissue with good biomechanics.	[25]
3D bioprinting	GF loaded on to polydopamine (PDA) nanoparticles (NPs) mixed with hydrogel		
FDM	PCL	Fibre-reinforced hybrid hydrogel structures allowed for a wider range of potential in hydrogels.	[26]
3D bioprinting	Cell-laden (C20A4) alginate hydrogel		
FDM	PLA	High cell proliferation rate and remained viability > 90% during the culture.	[24]
3D bioprinting	Gellan gum-poly (ethylene glycol) diacrylate (GG-PEGDA) double network hydrogel with murine bone marrow stromal cells.		
FDM (CEP, AF)	PCL	Compressive modulus was within the range of lumbar disc.	[27]
Hydrogel fill	hMSC cell-laden collagen-LMW HA-4S-Star_PEG_CNP hydrogel		
FDM	FlexiFil PLA (FPLA)	Scaffolds were stable, biocompatible, and allowed fibrocartilaginous matrix expression by MSCs and proteoglycan-rich ECM deposition by NP cells.	[28]
Hydrogel fill	Alginate hydrogel		
electrospinning	PLLA/POSS-(PLLA) nanofiber	A 6-month in vivo rat C3/C4 disc space implantation demonstrated maintenance of disc height and deposition of proteoglycan. Mechanical properties similar to that of native IVD.	[29]
FDM	PLA		
3D bioprinting	Gellan gum/polyethylene glycol diacrylate (GG/PEGDA) double network		
FDM (CEP, AF)	PCL	CTGF in AF region promoted fibrocartilage such as differentiation, and TGF-β3 in NP region promoted differentiation to hyaline cartilage-like cells. Bone marrow MSCs in IVD scaffold promotes Collagen type I deposition. TGF-β3 in NP region promoted deposition of glycosaminoglycans and collagen type II. CTGF in AF region promoted deposition of glycosaminoglycans and collagen type I.	[25]
3D bioprinting	Gelatin-hyaluronic acid-sodium alginate mixed with growth factors		
FDM	PLA	After 6 months implantation in rat C3/C4 space, scaffolds maintained their height and promoted deposition of proteoglycan and collagen.	[30]
3D bioprinting	GG-PEGDA hydrogel with rBMSC cells		
Selective laser sintering (SLS)	Polyurethane with modified “Bucklicrystal” structure	Showed appropriate mechanical behaviour along with in vitro and in vivo ability to restore physiological function.	[31,32]

## Data Availability

Not applicable.
